# The Toxicity Effects of Metformin and the Bioremediation of Metformin in Aquatic Plant Duckweed

**DOI:** 10.3390/plants14121761

**Published:** 2025-06-09

**Authors:** Bing Han, Yumeng Jiang, Wenqiao Wang, Yuhan Guo, Yunwen Yang, Yuman He, Qiqi Di, Ziyang Qu, Yun Xing, Lin Yang

**Affiliations:** 1 Tianjin Key Laboratory of Animal and Plant Resistance, College of Life Sciences, Tianjin Normal University, Tianjin 300387, China; 2 State Key Laboratory of Forage Breeding-by-Design and Utilization, Institute of Botany, Chinese Academy of Sciences, Beijing 100093, China; 3 Shanghai Key Laboratory of Regulatory Biology, Institute of Biomedical Sciences, School of Life Sciences, East China Normal University, Shanghai 200241, China

**Keywords:** *Lemna turionifera*, metformin, toxicology, accumulation

## Abstract

The release of metformin into the environment poses significant challenges, yet its effects on higher plants remain largely unexplored. In this study, we investigated the impact of metformin exposure on duckweed (*Lemna turionifera* 5511) across varying concentrations ranging from 0 to 0.16 mg/mL. Our findings revealed that leaves exhibited chlorosis, accompanied by a reduction in biomass, particularly evident at concentrations of 0.1, 0.13, and 0.16 mg/mL of metformin. Chlorophyll fluorescence analysis showed that MF exposure reduced photosynthetic performance, indicated by decreased Fv/Fm and Y (II), and increased Y (ND) and NPQ, suggesting impaired photosystem efficiency and altered energy dissipation. Additionally, genes involved in photosynthesis exhibited significantly reduced transcript abundance. Moreover, metformin was found to alter the transcript levels of GH3 and SAUR genes, which are associated with auxin signaling, and increase the expression of SnRK2, a key component of the abscisic acid signaling pathway. These findings shed light on the toxicological effects of metformin on higher plants, providing valuable evidence regarding the toxicity of this pharmaceutical contaminant. Subsequently, we investigated the absorption of metformin by duckweed (0.128 mg/g FW in 7 days) at a concentration of 0.13 mg/mL, observing a gradual decrease in metformin concentration to zero over a period of 10 days. Notably, the optimal adsorption time was determined to be ten days. Hence, duckweed emerges as a promising candidate for the concurrent bioremediation of metformin-contaminated water and the production of high-quality biomass.

## 1. Introduction

Metformin (MF) is the most widely used drug for treating type 2 diabetes [1], and it has applications in various fields, including anti-cancer [2,3], anti-aging [4] and weight reduction [5]. Due to its widespread use, MF has accumulated in significant quantities in aquatic organisms and the environment. In Australia, MF levels in wastewater range from 8.2 × 10^−6^ to 191 × 10^−4^ mg/mL, with a median concentration of 5.8 × 10^−5^ mg/mL [6]. In China, the mean concentrations of metformin in wastewater treatment plants vary from 2.42 × 10^−6^ mg/mL to 5.36 × 10^−5^ mg/mL [7]. It is worth noting that metformin can accumulate in biological tissues at concentrations much higher than those found in the surrounding environment [8,9]. These findings indicate that standard wastewater treatment processes are ineffective at eliminating this compound [10]. MF pollution adversely affects plant growth by inhibiting photosynthesis [11] and impairing the immune systems of animals and increasing the mortality of their larvae [12]. Therefore, assessing the toxicological effects of MF contamination in aquatic systems is of great significance, as is evaluating the efficiency of aquatic plants in mitigating these effects.

MF in aquatic environments has been shown to adversely affect both animals and plants. In medaka fish, MF exposure increased reactive oxygen species (ROS) in males, reduced glutathione (GSH) levels, and elevated catalase activity in females [13]. In *Chlorella vulgaris*, MF treatment led to increased non-photochemical quenching (NPQ), reduced electron transport rate, and impaired light energy utilization [14]. At the cellular level, MF enters mammalian cells via organic cation transporters (e.g., OCT1 and OCT2) [15], while in plants, it is likely taken up through roots and membrane transport proteins, though specific mechanisms remain unclear [16]. MF primarily targets mitochondria by inhibiting complex I of the respiratory chain, thereby activating the AMPK pathway [17], which also modulates immune responses in animals [18]. Given these widespread physiological and molecular effects, further investigation of MF toxicity and its environmental implications is critically important.

Aquatic plant species are under investigation to evaluate their capabilities and effectiveness in phytoremediation applications, particularly focusing on rapidly growing species such as duckweed [19]. Duckweed, a free-floating macrophyte, has garnered significant attention due to its high capacity for the adsorption and transfer of nitrogen, phosphorus, organic matter, and heavy metals [20], making it effective at purifying wastewater and restoring the environment [21]. As the smallest and simplest flowering plant in the world [22], duckweed consists of a flattened thallus and a simple root, allowing it to float on the water surface and absorb nutrients efficiently [23]. Its simple growth requirements, rapid growth rate, and tolerance to various wastewater conditions [24] contribute to its status as a key model organism in toxicology studies [22,25].

MF demonstrates considerable persistence in both soil and aquatic environments due to its high solubility and stability. In soils, it persists with reported half-lives of 2.7–15.5 days and can take up to 123 days to achieve 90% dissipation [26]. In aquatic systems, MF degradation occurs primarily through photolysis and microbial activity, with half-lives ranging from several days to a few weeks depending on water chemistry and microbial communities [27]. Although the effects of metformin exposure on duckweed remain unknown, MF exhibits high stability against hydrolysis and photolysis [28] and, as MF-HCl is the predominant form in pharmaceuticals, is readily water-soluble. Meanwhile, duckweed is one of the most effective plants for wastewater treatment [29], with reports indicating that it can absorb significant amounts of the heavy metal cadmium [30]. Thus, duckweed may be utilized to remediate metformin in the environment, significantly addressing the pollution issue. In our experiments, duckweed was adopted to study the effect of MF on higher plants. In addition, we support the use of duckweed for the phytoremediation of water bodies contaminated by metformin due to its ability to hyper-accumulate the metal in its tissues.

In this study, we aimed to undertake the following: (i) examine the effect of MF on duckweed growth and chlorophyll fluorescence; (ii) investigate changes in gene expression of duckweed related to photosynthesis, plant hormones, and carbon fixation under MF treatment; (iii) measure the adsorption capacity of duckweed for MF. This study provides new insights into the management of MF pollution in aquatic systems.

## 2. Results

### 2.1. MF Exposure Inhibited Duckweed Growth

**Figure 1:**
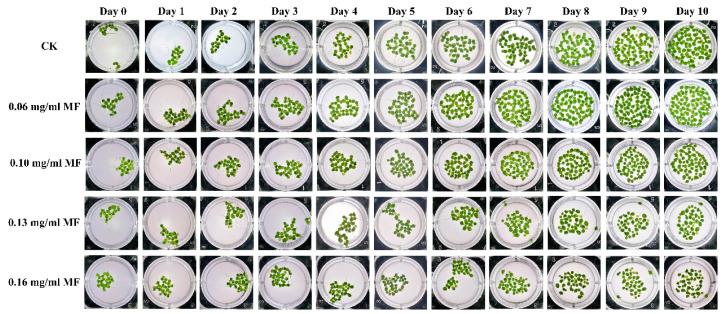
The phenotypes of the duckweed cultured under different MFs for 10 days. The concentrations for MFs were 0.06, 0.10, 0.13, and 0.16 mg/mL respectively.

The growth of duckweed exposed to varying concentrations of MF was observed over a ten-day period. As illustrated in Figure 1, morphological observations revealed chlorosis and atrophy in duckweed fronds, particularly in newly developed individuals. The overall size of duckweed fronds was significantly reduced, accompanied by varying degrees of chlorosis depending on MF concentration and exposure duration. Specifically, no noticeable changes in frond size or color were observed under 0.06 mg/mL MF treatment throughout the ten-day period. In contrast, at 0.10 mg/mL MF, a reduction in the size of newly developed fronds along with slight chlorosis was observed by day 10. Exposure to 0.13 mg/mL MF resulted in more pronounced effects, with chlorosis appearing by day 6 and most newly developed fronds exhibiting both yellowing and severe size reduction after ten days.

**Figure 2:**
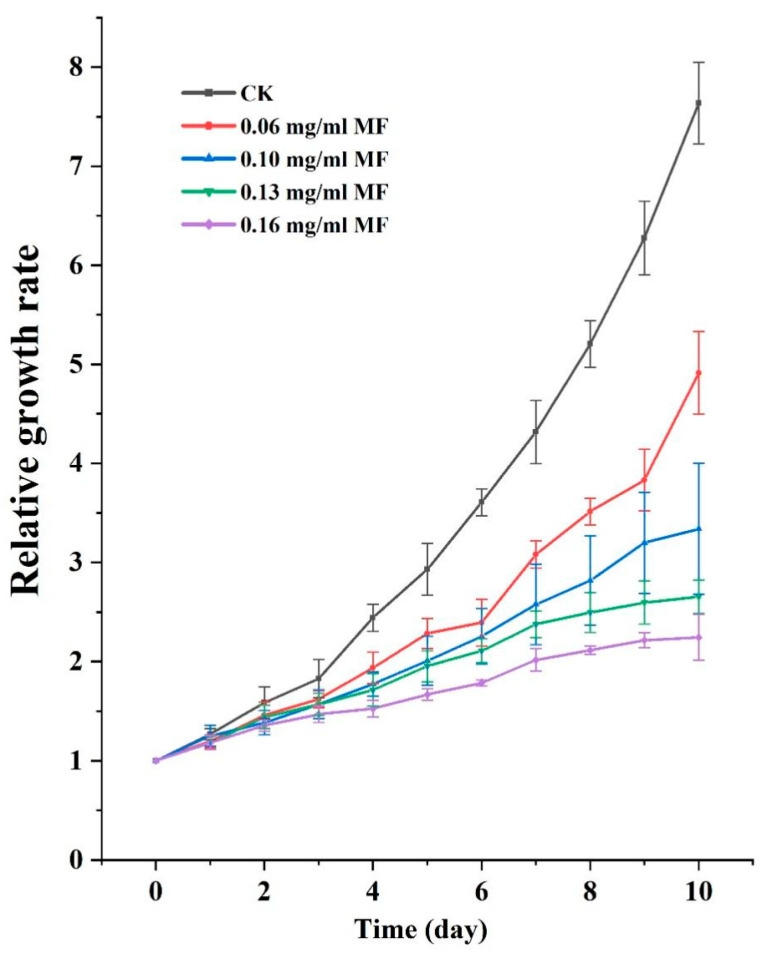
RGR of duckweed at different concentrations of MF over a 10-day exposure period. MF concentrations were 0.06, 0.10, 0.13, and 0.16 mg/mL, respectively. RGR was calculated as RGR = (number of frond clusters at time t)/(number of frond clusters at day 0), where t refers to the sampling day (e.g., day 1, 4, 7, or 10). Data are presented as means ± standard deviation (SD, *n* = 3).

As shown in Figure 2, MF exposure significantly inhibited the relative growth rate (RGR) of duckweed. Higher MF concentrations resulted in greater suppression of growth. At 0.06 mg/mL, duckweed exhibited a slight reduction in RGR during the early stages; however, recovery began on day 6, and the growth trajectory approached that of the control group by day 9. Under 0.10 mg/mL MF treatment, growth was comparable to the control within the first three days, but a marked decline in RGR was observed from day 4 onward. At 0.13 mg/mL, the inhibition was more pronounced: although the growth pattern initially resembled that of the control group during the first two days, RGR dropped sharply from day 3 and subsequently plateaued. A similar trend was observed at 0.16 mg/mL, with sustained suppression of RGR. Based on these results, 0.13 mg/mL MF was selected as the representative treatment concentration for subsequent experiments.

### 2.2. Photosynthetic Efficiency Was Affected by MF

**Figure 3:**
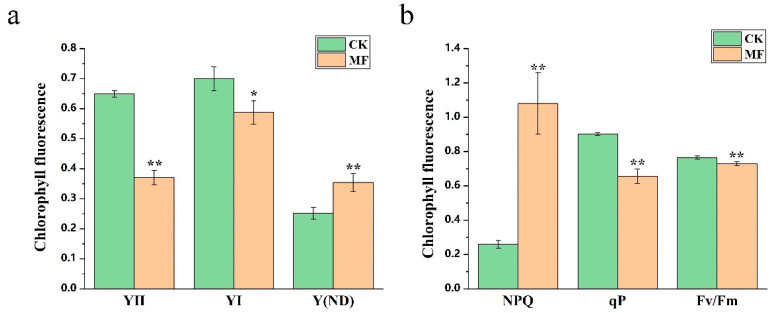
The chlorophyll fluorescence [YII, YI, Y (ND), NPQ, Fv/Fm, and qP] of duckweed fronds with or without 0.13 mg/mL MF treatment for 8 days. Data are shown as mean ± SD (*n* = 5). Statistical significance was determined using the Independent Samples *t*-test. Asterisks indicate significant differences between CK and MF: *p* < 0.05 (*) and *p* < 0.01 (**). (**a**) YII, YI, and Y(ND) reflect quantum yields related to PSI and PSII; (**b**) NPQ, qP, and Fv/Fm represent energy dissipation, photochemical efficiency, and maximum quantum yield of PSII, respectively.

Photosynthetic efficiency parameters, including Y (I), Y (II), Fv/Fm, qP, NPQ, and Y (ND), were analyzed in duckweed under MF exposure (Figure 3). High concentrations of MF markedly affected photosynthetic performance. Y (I), representing the effective quantum yield of PSI, and Y (II), representing the effective quantum yield of PSII, were significantly reduced under MF treatment, decreasing by 16% and 42.9%, respectively, compared to the control group (CK). Fv/Fm, indicating the maximum photochemical efficiency of PSII, decreased by 4.8% under MF exposure. qP, the photochemical quenching coefficient, was reduced by 27.3%. In contrast, NPQ, representing non-photochemical quenching, increased by 315.9%, suggesting enhanced thermal energy dissipation via the xanthophyll cycle, a protective mechanism helping to maintain photosynthetic stability.

Additionally, the increase in Y (ND) under MF exposure may reflect enhanced donor-side limitations, suggesting that duckweed experienced excess excitation energy that required dissipation. Overall, the transcriptional and chlorophyll fluorescence measurements suggested that MF exposure may adversely affect light energy utilization and electron transport efficiency in PSII.

### 2.3. The Expression of Critical Genes Involved in Photosynthesis and Photosynthesis—Antenna Pathways Were Downregulated Due to MF Exposure

**Figure 4:**
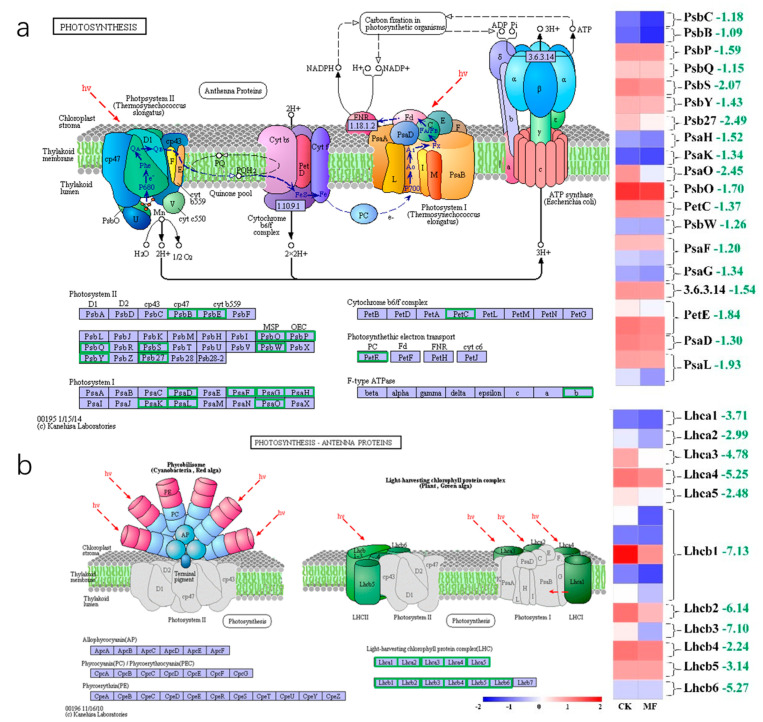
KEGG enrichment analysis of genes involved in photosynthesis (**a**) and photosynthesis-antenna proteins (**b**) in the “MF vs. CK” comparison. The green boxes indicate that the transcript levels of genes encoding the corresponding proteins were significantly downregulated after MF treatment. Normalization [log_2_(FPKM + 1)] and standardization (Z-score) were applied to plot a cluster heat map of all DEGs in both pathways, with red to blue representing high to low gene expression levels, respectively. Each group of DEGs corresponding to a specific protein is indicated by brackets, and the green numbers represent the average fold-change values of the associated unigenes at the transcriptomic level. Abbreviations: *PsbC*: photosystem II CP43 chlorophyll apoprotein; *PsbB*: photosystem II CP47 chlorophyll apoprotein; *PsbP:* photosystem II oxygen-evolving enhancer protein 2; *PsbQ*: photosystem II oxygen-evolving enhancer protein 3; *PsbS:* photosystem II 22 kDa protein; *PsbW*: photosystem II PsbW protein; *PsbY*: photosystem II PsbY protein; *Psb27*: photosystem II Psb27 protein; *PsaD*: photosystem I subunit II; *PsaF*: photosystem I subunit III; *Lhca1–5*: light-harvesting complex I chlorophyll a/b binding protein 1–5; *Lhcb1–6*: light-harvesting complex II chlorophyll a/b binding protein 1–6.

To investigate MF-induced effects on photosynthesis-related pathways, transcriptome analyses were performed under MF stress, and the expression of genes associated with Photosystem II (PSII) and Photosystem I (PSI) were analyzed (Figure 4a and Table S1). MF exposure downregulated PSII-related genes significantly. The expression of *psbC* and *psbB*, encoding internal antenna proteins CP43 and CP47, was significantly downregulated by 2.07 and 1.59 log_2_ fold changes, respectively. The expression levels of *psbP* and *psbQ* [31], involved in PSII assembly and repair, were also downregulated by 1.43 and 1.70 log_2_ fold changes. In addition, the expression levels of *psbW* and *psbY*, associated with PSII supercomplex stability, were reduced by 1.26 and 1.18 log_2_ fold changes. Under MF exposure, the transcript levels of *psaD* and *psaF* were downregulated by 1.30 and 1.20 log_2_ fold changes, respectively; both genes encode peripheral subunits of PSI. LHCI and LHCII, composed of chlorophyll a/b binding proteins known as light-harvesting complexes I (*Lhca*) and II (*Lhcb*), were downregulated by 2.48–5.25 and 2.24–7.13 log_2_ fold changes under MF stress, respectively. The results showed that MF exposure led to the downregulation of several *Lhca I*–*V* and *Lhcb I*–*VI* gene isoforms (Figure 4b and Table S2). Among them, LhcaI and LhcbII exhibited the most pronounced decreases in transcript levels. The altered expression of photosynthesis-related genes was consistent with the measured photosynthetic parameters.

### 2.4. Differentially Expressed Genes (DEGs) After MF Treatment

**Figure 5:**
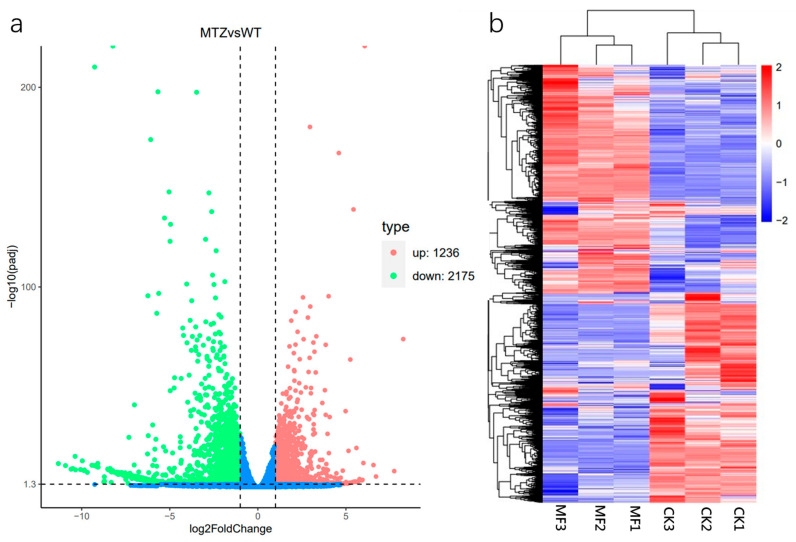
(**a**) A volcano map consisting of genes significantly upregulated and downregulated in “MF vs. CK”. The greater the absolute value of log_2_ fold change, the larger the fold change in gene expression. The larger the value of −log_10_ (padj), the more significant the differential expression between the “MF” and “CK”. The significance of differential expression was screened with a threshold of |log_2_ fold change| > 1 and −log_10_(padj) > 1.3. (**b**) A cluster analysis of total DEGs for each sample of “MF” and “CK”. Red color in the heat map meant high expression, while blue meant low expression.

To further investigate the molecular mechanisms underlying the effects of MF on duckweed growth, differentially expressed genes (DEGs) were analyzed and categorized. A total of 4291 DEGs were identified as duckweed, as shown in Figure 5a, including 1664 significantly upregulated genes and 2627 significantly downregulated genes. The overall expression profiles of these DEGs are illustrated in Figure 5b, where red indicates high expression levels and blue indicates low expression levels.

### 2.5. Gene Ontology and KEGG Pathway Analyses of DEGs

**Figure 6:**
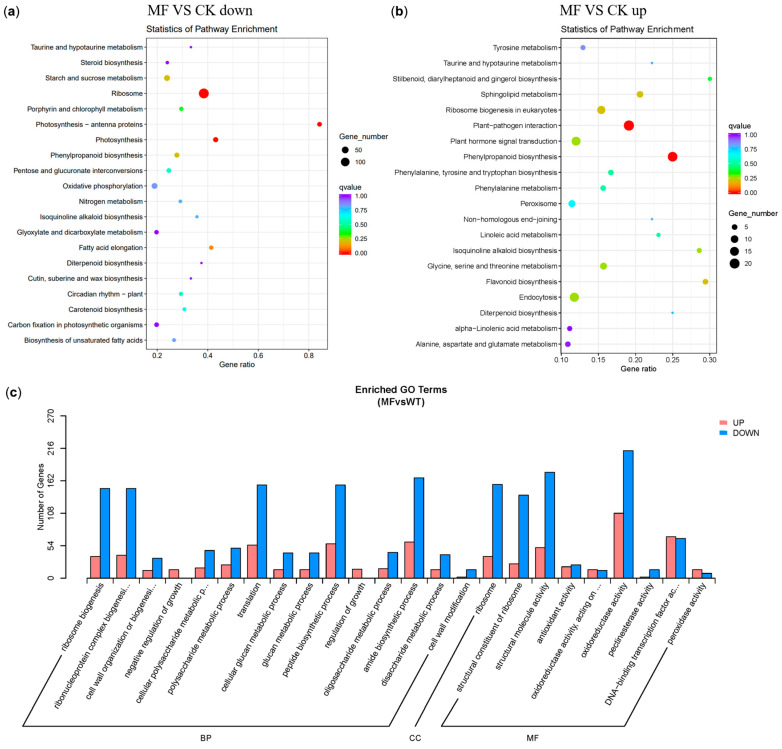
The top 20 KEGG pathways that were maximally downregulated (**a**) and upregulated (**b**) in the “MF vs. CK” comparison are presented. The gene ratio, defined as the ratio of differential genes in a pathway to the total number of annotated genes in that pathway, was used as the screening criterion. The q-value ranged from 0 to 1: the closer the value to zero, the more obvious the enrichment was, while the color also changes from purple to red. The q-value ranges from 0 to 1: the closer the value to zero, the more significant the enrichment, as indicated by a color gradient changing from purple to red. (**c**) For the “MF vs. CK” comparison, regarding the number of enriched DEGs in gene ontology categories, red meant upregulation and blue meant downregulation.

To investigate the changes in biochemical metabolic and signal transduction pathways associated with DEGs under MF exposure, KEGG enrichment analysis was conducted. As shown in Figure 6a, among the downregulated genes, the top-enriched was “Photosynthesis—antenna proteins” with 16 downregulated DEGs (Rich factor > 0.8) and a significant enrichment degree (q-value near”0”). It was followed by “Photosynthesis” and “Ribosome”, which had 22 and 143 downregulated DEGs, respectively, with a Rich factor around 0.4. In contrast, among the upregulated pathways, as shown in Figure 6b, the top-enriched pathways were “Phenylpropanoid biosynthesis” and “Plant-pathogen interaction”, with 16 and 21 upregulated DEGs, respectively. Although the degree of enrichment was significant, the enrichment ratio was low, with Rich factors of only about 0.25 and 0.2, respectively. In the photosynthesis-related DEGs, the transcript levels of *psbW*, *psaO*, *psaL*, and *psaH*, associated with PSII stability and PSI electron transfer, were significantly downregulated under MF exposure.

To further investigate the biological processes associated with MF-responsive DEGs, gene ontology (GO) enrichment analysis was performed. As shown in Figure 6c, most GO categories were enriched with genes exhibiting downregulated transcript levels, particularly those related to ribosome-associated processes, such as “ribosome biogenesis” and “ribonucleoprotein complex biosynthesis”, followed by “translation” and “structural molecular activity”.

### 2.6. MF Suppressed Photosynthetic Carbon Fixation

**Figure 7:**
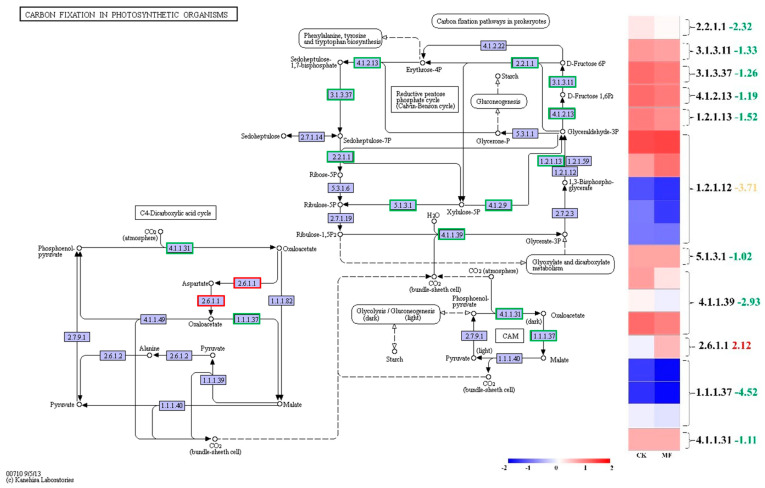
KEGG pathway analysis of carbon fixation in photosynthetic organisms comparing “MF vs. CK”. Rectangles represent enzyme abbreviations, with color coding based on the expression patterns of the corresponding genes: green indicates downregulation, red indicates upregulation, and yellow indicates mixed expression changes. A cluster heat map was generated based on normalized [log_2_(FPKM + 1)] and standardized (Z-score) expression values, where red indicates high expression and blue indicates low expression. Curly brackets group DEGs encoding the same enzyme, and the adjacent numbers represent the average log_2_fold change values of all the corresponding unigenes. Abbreviations: 2.2.1.1: transketolase; 3.1.3.11: fructose-1,6-bisphosphatase I; 3.1.3.37: sedoheptulose-bisphosphatase; 4.1.2.13: fructose-bisphosphate aldolase, class I; 1.2.1.13: glyceraldehyde-3-phosphate dehydrogenase (NADP+) (phosphorylating); 1.2.1.12: glyceraldehyde 3-phosphate dehydrogenase (phosphorylating); 5.1.3.1: ribulose-phosphate 3-epimerase; 4.1.1.39: ribulose-1,5-bisphosphate carboxylase; 2.6.1.1: aspartate aminotransferase, cytoplasmic; 1.1.1.37: malate dehydrogenase; 4.1.1.31: phosphoenolpyruvate carboxylase.

CO_2_ fixation is a crucial component of the photosynthetic process, primarily occurring through the Calvin cycle, which contains three stages: carboxylation, reduction, and regeneration. Ribulose-1,5-bisphosphate (Rubisco, EC 4.1.1.39) plays a central role in this process and is also a key enzyme in plant photorespiration. As shown in Figure 7 and Table S3, the transcript level of the gene encoding Rubisco was significantly downregulated under MF exposure, with a log_2_ fold change of 2.93. Additionally, five enzymes involved in the Calvin cycle also exhibited decreased transcript levels. These transcriptional changes suggest potential impacts on Calvin cycle activity; however, further physiological measurements would be necessary to confirm functional consequences on carbon fixation.

### 2.7. MF Modified Signal Transduction of Auxin and Abscisic Acid

**Figure 8:**
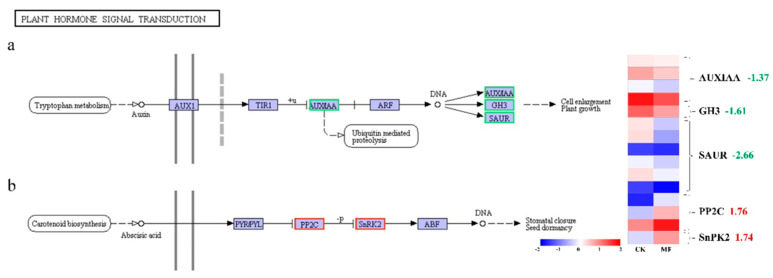
KEGG pathway analysis of auxin (**a**) and abscisic acid (ABA) (**b**) signal transduction pathways in the “MF vs. CK” comparison. Each box represents an abbreviated name of a signaling component, and the color reflects the expression pattern of the corresponding gene: green indicates downregulation and red indicates upregulation. A cluster heat map was generated based on normalized [log_2_(FPKM + 1)] and standardized (Z-score) expression values, with colors shifting from red to blue indicating the relative expression levels from high to low. Curly brackets group DEGs encoding the same signaling component, and the adjacent numbers indicate the average log_2_ fold change values of the associated genes. Abbreviations: AUXIAA: auxin-responsive protein IAA; *GH3*: auxin responsive GH3 gene family; *SAUR*: auxin responsive SAUR gene family; PP2C: protein phosphatase 2C; SnRK2: serine/threonine-protein kinase SRK2.

To further explore MF-induced molecular responses, DEGs related to phytohormone signal transduction were analyzed, with a focus on auxin and abscisic acid (ABA) pathways (Figure 8 and Table 1). In the auxin signaling pathway, transcript levels of *GH3* and *SAUR*, key downstream genes regulated by auxin response factors (ARFs), were downregulated following MF exposure, suggesting potential impacts on auxin-responsive gene expression. In contrast, the transcript levels of SnRK2, a positive regulator in ABA signaling, were upregulated under MF treatment. These transcriptional changes imply the possible modulation of hormone-related pathways; however, further physiological and biochemical validation is needed to confirm functional outcomes.

### 2.8. Analysis of MF Enrichment by Duckweed

**Figure 9:**
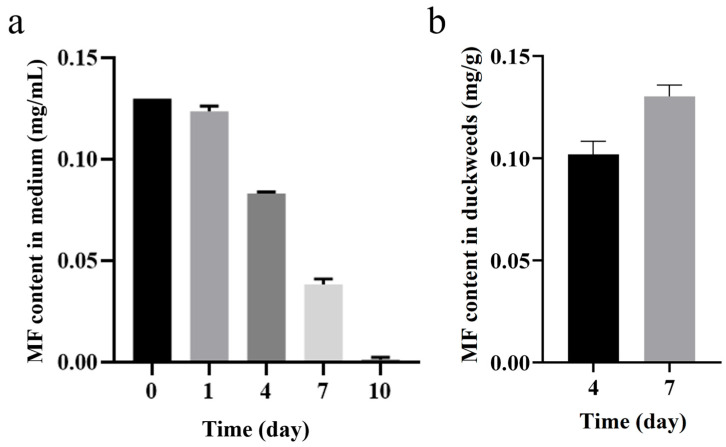
(**a**) A total of 0.13 mg/mL MF was exposed to duckweed, and MF concentrations changed at 0, 1, 4, 7, and 10 days, respectively. A total of 0.13 mg/mL MF was exposed to duckweed, and MF concentrations changed at 0, 1, 4, 7, and 10 days, respectively. (**b**) MF content in duckweed tissues at day 4 and day 7 after exposure to 0.13 mg/mL MF, expressed as milligrams per gram of fresh weight (mg/g FW).

The enrichment of MF by duckweed was continuously observed over 10 days. As shown in Figure 9a, when exposed to 0.13 mg/mL MF, the concentration of MF gradually decreased over time. On the first day, the concentration of MF declined to 0.123 mg/mL, followed by 0.083 mg/mL on the fourth day and 0.038 mg/mL on the seventh day. By the tenth day, the concentration had decreased to 0.001 mg/mL, nearly reaching zero. These results showed that duckweed exhibits a strong capacity for MF enrichment.

The concentration of MF in the culture medium was monitored over a 10-day period to evaluate its change after exposure to duckweed. As shown in Figure 9a, upon treatment with 0.13 mg/mL MF, the concentration gradually declined over time. Specifically, MF decreased to 0.123 mg/mL on day 1, 0.083 mg/mL on day 4, and 0.038 mg/mL on day 7. By day 10, the concentration had dropped to 0.001 mg/mL. In contrast, in the blank control group (the medium with MF but without duckweed), the MF concentration remained stable throughout the 10-day period.

To further investigate the interaction between MF and duckweed, the MF content in duckweed tissues was also quantified on days 4 and 7. As shown in Figure 9b, MF concentrations in duckweed were found to be 0.106 mg/g FW and 0.128 mg/g FW.

## 3. Discussion and Conclusions

### 3.1. Effects of Metformin on Duckweed Growth and Photosynthesis

The results of this study demonstrate that exposure to metformin (MF) significantly inhibited the growth of duckweed, with more pronounced detrimental effects observed at higher concentrations (0.13 mg/mL). Specifically, duckweed exhibited evident chlorosis and smaller newly developed fronds, leading to a significant reduction in biomass. These findings are consistent with previous studies on other aquatic plants, such as Chlorella vulgaris and Daucus carota [14]. The observed chlorosis and smaller fronds are indicative of inhibited growth rather than the shrinkage of existing fronds. Biomass reduction was due to slower growth rates rather than a decrease in overall biomass.

*PsbP*, *psbQ*, and *psbS* are involved in PSII assembly and repair, which contribute to photoprotection [31,32]. In addition, *psbW* and *psbY* are associated with PSII supercomplex stability [33,34], and *psb27* is required for PSII repair [35]. In our study, there are some functional impairment and gene expression changes with MF treatment: (1) Significant reductions in Y (II) (−42.9%) and qP (−27.3%) indicate impaired photochemical efficiency of PSII (Figure 3). (2) Elevated NPQ (+315.9%) and Y (ND) demonstrate enhanced energy dissipation due to electron transport bottlenecks (Figure 3). (3) These functional changes align with the structural destabilization of PSII (Fv/Fm ↓4.8%) and PSI (Y (I) ↓16%). (4) PSII core genes (psbB, psbC, psbP/Q) were downregulated correlates with the loss of antenna proteins (CP43/CP47), as well as the reduced expression of PSI subunits (*psaD*, *psaF*) and light-harvesting complexes (*Lhca*/*b*). These influences might have led to light capture integrated transcriptomic and chlorophyll fluorescence analyses indicating that MF exposure reduces photosynthetic efficiency by downregulating critical genes.

These observations indicate that MF not only impairs the effective utilization of light energy in the photosystem but also enhances the dissipation of excess energy as a protective response against stress-induced damage. Additionally, RNA-seq analysis showed significant downregulation of genes related to photosynthesis and exhibited reduced transcript levels under MF stress. These findings provide substantial evidence to help us better understand the phytotoxic effects of MF on plants.

### 3.2. Effects of Metformin on Plant Hormone Signal Transduction in Duckweed

Our analyses revealed that MF treatment led to the downregulation of key genes involved in auxin signaling pathways (*GH3* and *SAUR*), implying the suppression of auxin signaling. Conversely, the expression of *SnRK2*, a crucial gene in the abscisic acid (ABA) signaling pathway, was significantly upregulated. Previous studies have indicated that reduced auxin signaling typically results in the inhibition of plant growth, whereas enhanced ABA signaling can activate defensive mechanisms, thereby improving stress tolerance in plants [36,37]. Thus, MF may coordinately regulate these two hormone signaling pathways, consequently affecting both plant growth and stress responses.

### 3.3. Potential of Duckweed in Remediation of Metformin-Contaminated Water

Our study confirms the capability of duckweed to accumulate metformin from contaminated water (Figure 9). MF concentration in the medium decreased from 0.13 mg/mL to near-zero (Figure 9a), with MF detected in plant tissues (0.106–0.128 mg/g FW) after 7 days of treatment (Figure 9b). This result demonstrates strong accumulation potential for this pharmaceutical contaminant. However, the specific metabolic and transformation pathways after duckweed’s uptake of MF have not been deeply investigated in this study. Future research should focus on determining whether duckweed converts accumulated MF into less toxic or non-toxic products and elucidating the detailed metabolic mechanisms involved.

In conclusion, this study reveals the inhibitory mechanisms of MF on duckweed growth and photosynthesis, elucidates the molecular mechanism of duckweed’s response to MF stress via hormone signaling regulation, and confirms the ecological remediation potential of duckweed for MF-contaminated environments. Future work should explore the long-term effects of low-concentration MF exposure and the metabolic fate of MF within duckweed tissues.

## 4. Materials and Methods

### 4.1. Culture of Duckweed and Metformin (MF) Exposure

The duckweed clone used in this study (*Lemna turionifera* 5511) was originally collected from Xiqing Lake in Tianjin, China. While initially misidentified as *Lemna minor* in our earlier work, subsequent molecular characterization through PCR amplification, sequencing (Illumina NovaSeq 6000, Illumina, San Diego, CA, USA), and BLAST (NCBI online version) alignment confirmed its taxonomic identity as *Lemna turionifera* in 2017 [38] (Figures S1 and S2). This clone has been continuously maintained in our laboratory collection through asexual propagation since 2005, with periodic verification of genetic stability.

Prior to the experiments, the duckweed was washed with deionized water, disinfected with 15% NaClO for 10 min, and then rinsed with sterile water 3–5 times. Subsequently, the duckweed was cultivated in a liquid medium known as DATKO [39,40]. The cultivation conditions were maintained at a temperature of 26 °C during the day and 20 °C at night, with a 16 h light and 8 h dark cycle, and a light intensity of 95 μmol m^−2^ s^−1^.

Based on a preliminary concentration experiment, 0.13 mg/mL was selected as the optimal MF treatment concentration for subsequent physiological and molecular analyses. A stock solution of MF was prepared at 13 mg/mL, and 100 μL of this solution was added to each well of a six-well plate containing 10 mL of DATKO medium to achieve a final concentration of 0.13 mg/mL. An equal number of duckweed frond clusters were carefully transferred into each well. The treatment lasted for 48 h under the same environmental conditions as cultivation.

### 4.2. Determination of the Growth Curves of Duckweed Under MF Treatment

The duration for which the duckweed was treated with or without MF was 10 days, with observations recorded daily at the same time. The concentrations of MF were 0, 0.06, 0.10, 0.13, and 0.16 mg/mL. These concentrations were used to evaluate the effects of MF on duckweed growth. Growth curves were plotted according to the RGR of the duckweed. The RGR was calculated by measuring the number of fronds per day by the initial number of fronds. RGR = (number of frond clusters at time t)/(number of frond clusters at day 0), where t refers to the sampling day (e.g., day 1, 4, 7, or 10).

### 4.3. Measurement of Photosynthetic Fluorescence Parameters

The duckweed was treated with or without 0.13 mg/mL of MF for 7 days. Initially, the duckweed were taken for 30 min of dark adaptation. Subsequently, the actual quantum yield (YII), the global quantum yield of PSI (YI), the maximum quantum yield (Fv/Fm), the photochemical quenching coefficient (qP), the non-photochemical quenching coefficient (Y (ND)), and the NPQ of duckweed leaves were measured using the Dual-PAM100 fluorometer (Walz GmbH, Effeltrich, Germany) at the same time as the morphological index. After that, a fast-light curve measurement was performed. Ten successive increasing intensities of photochemical light (30 s each), i.e., 14, 22, 40, 98, 176, 218, 334, 505, 763, and 1182 μ mol photons m^−2^ s^−1^, were provided sequentially to the leaves of duckweeds.

### 4.4. RNA Extraction and Sequencing

Total RNA was extracted using a standard TRIzol-based protocol ((Invitrogen, Carlsbad, CA, USA) following the manufacturer’s instructions. The quality and concentration of RNA were first assessed using a Qubit 2.0 Fluorometer (Thermo Fisher Scientific, Waltham, MA, USA), followed by integrity evaluation using an Agilent 2100 Bioanalyzer (Agilent Technologies, Santa Clara, CA, USA). Only RNA samples with an RNA Integrity Number (RIN) greater than 7.0 were used for library construction.

For transcriptome library preparation, mRNA was purified from total RNA using poly-T oligo-attached magnetic beads. Fragmentation was performed with divalent cations under elevated temperature in First-Strand Synthesis Reaction Buffer (5×). First-strand cDNA was synthesized using random hexamer primers and M-MuLV Reverse Transcriptase (New England Biolabs, Ipswich, MA, USA), followed by second-strand synthesis using DNA Polymerase I (New England Biolabs, Ipswich, MA, USA) and dNTPs (Takara, Shiga, Japan). Overhangs were converted to blunt ends, and after 3′-adenylation, adaptors with hairpin loop structures were ligated. cDNA fragments of approximately 370–420 bp were selected using the AMPure XP system (Beckman Coulter, Brea, CA, USA), PCR-amplified, and purified to obtain the final libraries.

Library quality was validated by Qubit quantification and fragment-size detection using an Agilent 2100 Bioanalyzer. Quantitative PCR(CFX96 Real-Time PCR Detection System, Bio-Rad, Hercules, CA, USA) was used to determine the effective library concentration (>2 nM). Qualified libraries were pooled and sequenced on the Illumina NovaSeq 6000 platform (Illumina, San Diego, CA, USA), generating 150 bp paired-end reads via sequencing-by-synthesis.

Raw reads were processed to remove adaptors, low-quality reads, and reads with poly-N using in-house Perl scripts. Quality metrics such as Q20, Q30, and GC content were calculated. All subsequent analyses were performed using high-quality clean reads. Since no complete reference genome is available for *Lemna turionifera*, a de novo transcriptome assembly approach was adopted. Clean reads were assembled using Trinity v2.6.6 with default parameters except min_kmer_cov, which was set to 2 [41]. Assembly completeness was evaluated using BUSCO v3 with the Embryophyta database [42].

To reduce transcript redundancy, Corset v4.6 was used to cluster assembled transcripts based on shared read information across samples [43]. Functional annotation of unigenes was performed against multiple public databases: Nr (NCBI non-redundant protein), Nt (NCBI non-redundant nucleotide), Pfam, KOG/COG, Swiss-Prot, GO (gene ontology), and KEGG (Kyoto Encyclopedia of Genes and Genomes).

Differential expression was analyzed using DESeq2 v1.20.0 [44] for samples with replicates and edgeR v3.22.5 [45] for samples without replicates. Genes with padj < 0.05 and |log_2_(fold change)| > 1 were considered significantly differentially expressed. GO enrichment was conducted using GOseq v1.10.0 [46], and KEGG enrichment using KOBAS v2.0.12 [47], based on the hypergeometric distribution.

### 4.5. Determination of MF Enrichment by Duckweed

To evaluate the degradation and uptake of MF by duckweed, plants were evenly distributed in six-well plates to fully cover each well. MF was added to the medium at a final concentration of 0.13 mg/mL. At 1, 4, 7, and 10 days, 1 mL of culture solution was collected and transferred to a quartz cuvette for analysis. Absorbance at 232 nm was measured using a UV–Vis spectrophotometer (Shanghai INESA Scientific Instrument Co., Ltd., Anting, Shanghai, China), and MF concentrations were calculated based on a standard calibration curve (y = 0.6391x + 0.0007, R^2^ = 0.9955). All measurements were conducted in triplicate.

MF accumulation in duckweed tissues was quantified on days 4 and 7. Fresh samples (0.1 g) were placed in 1.5 mL Eppendorf tubes with 1 mL of ultrapure water and subjected to ultrasonic bath treatment (30 s per cycle, 10 cycles). The resulting extract was diluted 16-fold, and absorbance at 232 nm was measured using the same spectrophotometric method. Extracts from untreated duckweed were used as blanks for baseline correction. MF concentrations were determined using the established standard curve.

### 4.6. Statistical Analysis

All data are presented as mean ± standard deviation (SD) based on three biological replicates (*n* = 3). Statistical analysis was performed using the Independent Samples *t*-test with SPSS software (IBM SPSS Statistics, Version 26). Statistical significance was defined as *p* < 0.05 or *p* < 0.01 and is indicated by asterisks in the figures: *p* < 0.05 (*) and *p* < 0.01 (**).

## Figures and Tables

**Table 1:** Gene expression in auxin and abscisic acid signal transduction.

Description	Gene_Id	MF_ Readcount	CK_ Readcount	log_2_Fold Change	Pval	Padj
auxin-responsive protein IAA	Cluster-3667.11150	110.05	226.59	−1.04	3.21 × 10^−6^	3.07 × 10^−5^
auxin-responsive protein IAA	Cluster-3667.3607	189.65	499.70	−1.40	4.14 × 10^−11^	8.50 × 10^−10^
auxin-responsive protein IAA	Cluster-3667.2138	41.97	155.55	−1.89	5.77 × 10^−6^	5.27 × 10^−5^
auxin-responsive protein IAA	Cluster-3667.8895	1082.36	2400.65	−1.15	7.25 × 10^−14^	2.03 × 10^−12^
auxin responsive GH3 gene family	Cluster-3667.9729	349.46	1069.31	−1.61	8.22 × 10^−13^	2.07 × 10^−11^
SAUR family protein	Cluster-6625.0	38.64	240.96	−2.64	3.05 × 10^−19^	1.36 × 10^−17^
SAUR family protein	Cluster-3667.1437	21.11	260.06	−3.64	9.24 × 10^−36^	1.15 × 10^−33^
SAUR family protein	Cluster-6287.0	3.36	18.92	−2.45	0.003364	0.016163
SAUR family protein	Cluster-3667.19491	42.41	148.76	−1.81	2.08 × 10^−10^	3.89 × 10^−9^
SAUR family protein	Cluster-3667.14101	69.64	263.02	−1.92	2.16 × 10^−11^	4.59 × 10^−10^
SAUR family protein	Cluster-3667.17453	1.70	18.90	−3.49	0.000268	0.001697
protein phosphatase 2C	Cluster-3667.5265	55.00	13.81	2.00	1.06 × 10^−5^	9.17 × 10^−5^
protein phosphatase 2C	Cluster-3667.11703	257.77	87.59	1.56	2.89 × 10^−6^	2.78 × 10^−5^
protein phosphatase 2C	Cluster-3667.9131	2255.85	685.34	1.72	4.38 × 10^−12^	1.01 × 10^−10^
serine/threonine-protein kinase SRK2	Cluster-3667.6959	371.62	110.84	1.74	9.69 × 10^−12^	2.16 × 10^−10^

## Data Availability

RNA-Seq datasets supporting the findings of this study have been deposited in the NCBI Sequence Read Archive (SRA) under BioProject accession number PRJNA1263417 and are publicly accessible at the following link: https://dataview.ncbi.nlm.nih.gov/object/PRJNA1263417?reviewer=ukbqi9q2hl6a7fjj92eklcfee3. Accessed on 30 June 2026.

## Supplementary Materials

The following supporting information can be downloaded at https://www.mdpi.com/article/10.3390/plants14121761/s1, Molecular Identification of Duckweed; Table S1: Gene expression in photosynthesis; Table S2: Gene expression in photosynthesis-antenna protein; Table S3: Gene expression in carbon fixation in photosynthetic organisms. Figure S1: PCR products of psbK-psbI (1) and atpF-atpH (2); Figure S2: Sequence alignment of psbK-psbI and atpF-atpH.
