# Supplementary material for: The Toxicity Effects of Metformin and the Bioremediation of Metformin in Aquatic Plant Duckweed
Source: Plants (Basel). 2025 Jun 9;14(12):1761. doi: 10.3390/plants14121761 (PMC12196854; doi:10.3390/plants14121761)
Supplement: Supplementary file 1 [file plants-14-01761-s001.zip › plants-3552951-supplementary.pdf]

Molecular Identification of Duckweed

To confirm the species identity of the duckweed used in this study, total DNA was extracted from duckweed samples using standard CTAB-based protocols. Briefly, ~0.5 g fresh tissue was ground on ice, mixed with STES and TE buffers, followed by centrifugation, SDS treatment, phenol extraction, isopropanol precipitation, ethanol washing, and resuspension in ddH_2O. PCR amplification was performed using psbK-psbI primers

(forward: 5′ -TTAGCATTTGTTTGGCAAG-3′ ;

reverse: 5′ -AAAGTTTGAGAGTAAGCAT-3′)

and atpF-atpH primers

(forward:5′ -ACTCGCACACACTCCCTTTCC-3′ ;

reverse: 5′ -GCTTTTATGGAAGCTTTAACAAT-3′).

The PCR system (20 μl) included Premix Taq (Takara), template DNA, primers, and ddH_2O. Cycling conditions were: 95°C for 5 min; 30 cycles of 95°C 40 s, 56°C 1 min, 72°C 40 s; final extension at 72°C for 10 min. PCR products were verified by 1% agarose gel electrophoresis (Figure S1) and sequenced by Beijing Genomics institution. The sequences (476 bp for psbK-psbI, 643 bp for atpF-atpH) were aligned using NCBI BLAST (http://epigenome.rutgers.edu/cgi-bin/duckweed/blast.cgi). Both marker sequences showed 100% identity to Lemna turionifera, confirming the species used in this study. Sequence alignment screenshots are provided in Figure S2.

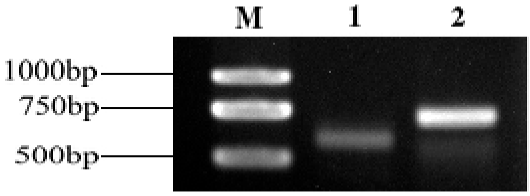

Figure S1 PCR products of psbK-psbI (1) and atpF-atpH (2)

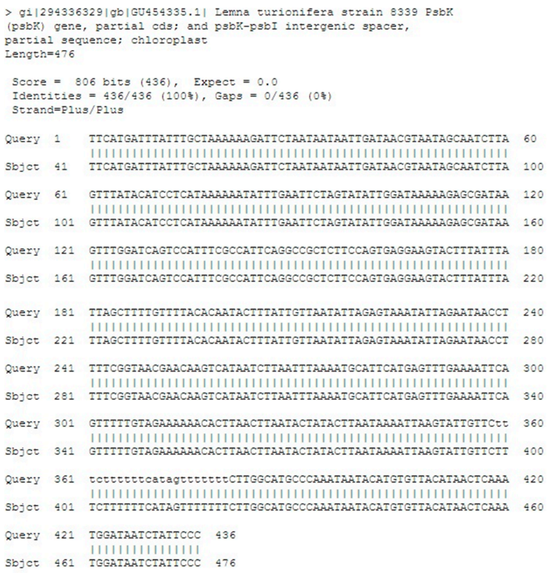

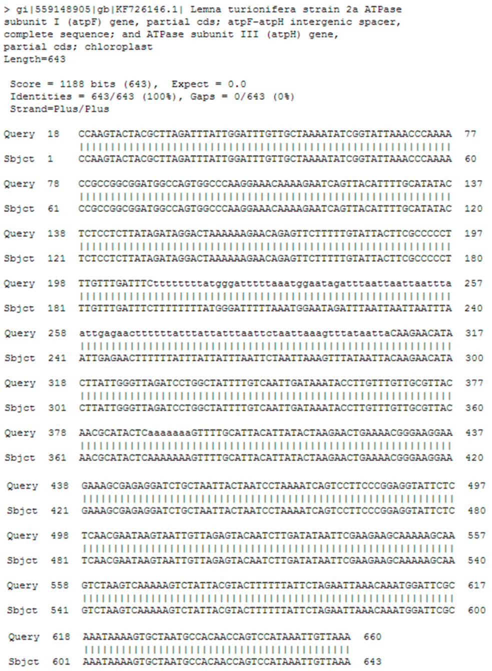

Figure S2 Sequence alignment of psbK-psbI and atpF-atpH

Table S1. Gene expression in photosynthesis.

Description	gene_id	MF_ readcount	WT_ readcount	log_2FoldChange	pval	padj
photosystem II CP43 chlorophyll apoprotein	Cluster-3667.972	51.83	116.96	-1.18	8.03E-05	0.00057351
photosystem II CP47 chlorophyll apoprotein	Cluster-3667.2476	40.38	86.21	-1.09	0.0019336	0.0099035
Cytochrome b6-f complex iron-sulfur subunit	Cluster-3667.8573	21300.81	54903.53	-1.37	3.24E-22	1.79E-20
photosystem II oxygen-evolving enhancer protein 1	Cluster-3667.8747	36247.81	118044.60	-1.70	1.82E-25	1.28E-23
photosystem II oxygen-evolving enhancer protein 2	Cluster-3667.8270	17189.34	51866.13	-1.59	2.88E-15	9.03E-14
photosystem II oxygen-evolving enhancer protein 3	Cluster-3667.7915	7145.38	15814.91	-1.15	2.88E-06	2.78E-05
photosystem II 22kDa protein	Cluster-3667.8614	18850.39	79031.84	-2.07	1.43E-30	1.34E-28
photosystem II PsbW protein	Cluster-3667.10414	9894.38	23621.46	-1.26	1.14E-12	2.81E-11
photosystem II PsbY protein	Cluster-3667.8509	9730.38	26142.31	-1.43	5.72E-17	2.05E-15
photosystem II Psb27 protein	Cluster-3667.7419	3027.66	17045.72	-2.49	3.59E-70	1.71E-67
plastocyanin	Cluster-3667.8599	25905.65	71850.78	-1.47	9.16E-21	4.66E-19
plastocyanin	Cluster-3667.11301	619.07	2843.11	-2.20	5.20E-15	1.60E-13
photosystem I subunit II	Cluster-3667.8105	19790.73	48821.62	-1.30	5.70E-11	1.14E-09
photosystem I subunit III	Cluster-3667.7841	10891.84	28621.87	-1.39	1.26E-11	2.77E-10
photosystem I subunit III	Cluster-3667.7158	5340.82	10693.64	-1.00	3.28E-07	3.79E-06
photosystem I subunit V	Cluster-3667.9596	8978.22	22778.40	-1.34	7.79E-15	2.36E-13
photosystem I subunit VI	Cluster-3667.8365	7314.89	20998.98	-1.52	1.19E-34	1.36E-32
photosystem I subunit X	Cluster-3667.9770	5723.61	14505.76	-1.34	5.36E-23	3.24E-21

photosystem I subunit XI	Cluster-3667.6172	2287.67	15971.15	-2.80	0.00010307	0.00071761
photosystem I subunit XI	Cluster-3667.8092	10690.24	22207.90	-1.05	1.91E-13	5.13E-12
photosystem I subunit PsaO	Cluster-3667.7698	11557.83	63223.11	-2.45	8.63E-50	2.06E-47
F-type H+-transporting ATPase subunit b	Cluster-3667.7621	22429.03	65094.19	-1.54	6.28E-25	4.25E-23

Table S2. Gene expression in photosynthesis- antenna protein.

Description	gene_id	MF_ readcount	CK_ readcount	log_2FoldChange	pval	padj
light-harvesting complex I chlorophyll a/b binding protein 1	Cluster-3667.12425	290.95	3818.29	-3.71	4.67E-71	2.27E-68
light-harvesting complex I chlorophyll a/b binding protein 2	Cluster-3667.11722	1173.67	9317.82	-2.99	2.28E-80	1.38E-77
light-harvesting complex I chlorophyll a/b binding protein 3	Cluster-3667.9127	2092.96	57532.56	-4.78	2.18E-108	2.93E-105
light-harvesting complex I chlorophyll a/b binding protein 4	Cluster-3667.6446	3979.43	151355.97	-5.25	8.75E-29	7.59E-27
light-harvesting complex I chlorophyll a/b binding protein 5	Cluster-3667.12078	623.31	3481.86	-2.48	1.33E-63	4.99E-61
light-harvesting complex II chlorophyll a/b binding protein 1	Cluster-3667.4534	93.71	29234.79	-8.29	2.99E-49	7.03E-47
light-harvesting complex II chlorophyll a/b binding protein 1	Cluster-3667.7080	124.07	6362.07	-5.68	2.00E-110	3.11E-107
light-harvesting complex II chlorophyll a/b binding protein 1	Cluster-3667.12909	0.00	72.82	-8.90	1.58E-12	3.83E-11
light-harvesting complex II chlorophyll a/b	Cluster-3667.7081	3775.46	478707.83	-6.99	3.28E-44	6.23E-42

binding protein 1						
light-harvesting complex II chlorophyll a/b binding protein 1	Cluster-3667.15303	72.66	6832.65	-6.55	1.22E-20	6.09E-19
light-harvesting complex II chlorophyll a/b binding protein 1	Cluster-3667.11549	353.83	29766.36	-6.39	7.23E-35	8.45E-33
light-harvesting complex II chlorophyll a/b binding protein 3	Cluster-3667.13361	278.15	38010.75	-7.09	1.06E-132	2.86E-129
light-harvesting complex II chlorophyll a/b binding protein 4	Cluster-3667.8866	24812.97	116955.74	-2.24	7.57E-59	2.52E-56
light-harvesting complex II chlorophyll a/b binding protein 5	Cluster-3667.8232	7001.30	61577.54	-3.14	8.57E-78	4.97E-75
light-harvesting complex II chlorophyll a/b binding protein 6	Cluster-3667.12138	450.26	17329.16	-5.27	3.06E-84	2.01E-81

Table S3. Gene expression in carbon fixation in photosynthetic organisms.

Description	gene_id	MF_ readcount	CK_ readcount	log_2FoldChange	pval	padj
transketolase	Cluster-3667.11660	125.72	625.63	-2.32	1.33E-18	5.52E-17
fructose-1,6-bisphosphatase I	Cluster-3667.9430	13484.05	33961.67	-1.33	4.20E-20	2.00E-18
sedoheptulose-bisphosphatase	Cluster-3667.8426	17988.36	43105.81	-1.26	1.82E-14	5.33E-13
fructose-bisphosphate aldolase, class I	Cluster-3667.8566	49535.50	112967.38	-1.19	5.08E-11	1.03E-09
glyceraldehyde-3-phosphate dehydrogenase (NADP+) (phosphorylating)	Cluster-3667.8148	24684.35	70875.68	-1.52	1.68E-21	8.95E-20
glyceraldehyde 3-phosphate dehydrogenase (phosphorylating)	Cluster-3667.8466	23188.03	11264.56	1.04	1.53E-11	3.34E-10
glyceraldehyde 3-phosphate dehydrogenase (phosphorylating)	Cluster-3667.8114	7838.08	2773.41	1.50	8.12E-18	3.14E-16
glyceraldehyde 3-phosphate	Cluster-1208.0	0.00	25.41	-7.38	2.68E-06	2.59E-05

dehydrogenase (phosphorylating)						
glyceraldehyde 3-phosphate dehydrogenase (phosphorylating)	Cluster-1206.0	0.00	12.00	-6.29	0.000136	0.000921
glyceraldehyde 3-phosphate dehydrogenase (phosphorylating)	Cluster-1513.1	0.28	51.60	-7.44	0.0082	0.034906
ribulose-phosphate 3-epimerase	Cluster-3667.8292	12808.85	25944.36	-1.02	8.88E-10	1.53E-08
ribulose-bisphosphate carboxylase small chain	Cluster-3667.8861	1588.83	28850.98	-4.18	2.06E-67	8.96E-65
ribulose-bisphosphate carboxylase small chain	Cluster-3667.15721	332.26	2927.49	-3.14	1.40E-57	4.51E-55
ribulose-bisphosphate carboxylase small chain	Cluster-3667.8434	40650.39	113118.50	-1.48	6.39E-10	1.12E-08
aspartate aminotransferase, cytoplasmic	Cluster-3667.7191	6729.81	1552.99	2.12	4.76E-47	9.98E-45
malate dehydrogenase	Cluster-1222.0	0.00	7.96	-5.70	0.003856	0.018213
malate dehydrogenase	Cluster-989.0	0.00	5.59	-5.19	0.007092	0.030726
malate dehydrogenase	Cluster-3667.6900	209.29	1334.63	-2.67	1.19E-38	1.79E-36
phosphoenolpyruvate carboxylase	Cluster-3667.7140	9416.50	20382.43	-1.11	7.08E-14	1.98E-12
